# A Repeated Measures Pilot Comparison of Trajectories of Fluctuating Endogenous Hormones in Young Women with Traumatic Brain Injury, Healthy Controls

**DOI:** 10.1155/2019/7694503

**Published:** 2019-02-12

**Authors:** Janet P. Niemeier, Paul B. Perrin, Bradley S. Hurst, David M. Foureau, Toan T. Huynh, Susan L. Evans, Jonathan E. Silverman, M. Elise McClannahan, Benjamin D. Brusch, Mark Newman, Jean-Luc Mougeot, Amy K. Wagner

**Affiliations:** ^1^Carolinas Medical Center, Charlotte, NC, USA; ^2^Department of Psychology, Virginia Commonwealth University, Richmond, VA, USA; ^3^Department of Physical Medicine and Rehabilitation, University of Pittsburgh, PA, USA

## Abstract

**Objective:**

To compare baseline and 72-hour hormone levels in women with traumatic brain injury (TBI) and controls.

**Setting:**

Hospital emergency department.

**Participants:**

21 women ages 18-35 with TBI and 21 controls.

**Design:**

Repeated measures.

**Main Measures:**

Serum samples at baseline and 72 hours; immunoassays for estradiol (E2), progesterone (PRO), luteinizing hormone (LH), follicle-stimulating hormone (FSH), and cortisol (CORT); and health history.

**Results:**

Women with TBI had lower E2 (*p* = 0.042) and higher CORT (*p* = 0.028) levels over time. Lower Glasgow Coma Scale (GSC) and OCs were associated with lower FSH (GCS *p* = 0.021; OCs *p* = 0.016) and higher CORT (GCS *p* = 0.001; OCs *p* = 0.008).

**Conclusion:**

Acute TBI may suppress E2 and increase CORT in young women. OCs appeared to independently affect CORT and FSH responses. Future work is needed with a larger sample to characterize TBI effects on women's endogenous hormone response to injury and OC use's effects on post-TBI stress response and gonadal function, as well as secondary injury.

## 1. Introduction

Researchers report mixed clinical findings in associating sex with multiple outcomes after traumatic brain injury (TBI) [1–10]. Both animal and human models of TBI have produced mixed findings related to sex hormone associations with post-TBI outcome domains and treatment effects [11–19]. Based on this work, equal numbers report women and men recover better than the opposite sex after TBI, and still others report no sex differences in recovery. Neuropsychological test performance studies show adult women outperforming men in executive functions, as well as verbal and visual memory after TBI [7, 8, 20–22]. However, these reported sex differences partially reflect well-documented sex differences in innate abilities of healthy children and adults [23–25]. In addition, rater bias related to gender-role expectations [26] and differential access to TBI rehabilitation for women after TBI may contribute to differing sex-based, post-TBI recovery trajectories [27, 28].

Evidence from experimental TBI models pointed toward progesterone and estradiol as providing neuroprotective effects for (primarily young male) rodents, findings which have supported inferences made in some reports of a neuroprotective advantage for women post-TBI [11, 13, 15]. In contrast, clinical TBI studies have shown that higher levels of sex hormones at time of injury, especially when TBI is severe, are markers for mortality or unfavorable outcomes in men and women [16, 29], particularly among older individuals with TBI. The results of two large multisite randomized, controlled trials administering progesterone during acute TBI were negative, despite significant and compelling evidence from experimental models demonstrating clear neuroprotective effects. Although phase II study findings supported progesterone as neuroprotective [17, 19], both multiphase III trials were stopped for futility [14, 30]. However, no sex hormone treatment studies have investigated if/how injury-induced steroidogenesis and/or age may influence treatment response to progesterone in a clinical population with TBI, despite findings that elevated progesterone mediates increased endogenous levels observed in both serum and CSF cortisol as well as serum estradiol and testosterone among individuals with severe TBI [16, 31], each of which is associated with poor outcomes.

Oral contraceptive (OC) use has been evaluated for possible beneficial effects on recovery after mild TBI. For example, female athletes taking OCs were less likely to report postconcussion symptoms [32, 33]. Also, two clinical TBI studies report additional findings which suggest greater heterogeneity of endocrine and neurophysiological processes for women after TBI. First, Wunderle et al. [18] found that women injured during the follicular phase, or who were taking OCs, reported higher quality of life outcomes than women injured during the luteal phase. Ranganathan et al. [12] collected multiple serum samples over time to compare post-TBI sex and stress hormone profiles of pre- and postmenopausal women with severe TBI. Results showed elevated cortisol levels were associated with anovulation and central hypothalamic-pituitary-ovarian (HPG) axis suppression. Further, resolution of elevated cortisol levels corresponded with resolution of suppressed luteinizing hormone (LH) and resumption of menses.

Multiple investigators have called for further, more rigorous research to determine if and how hormones influence recovery [18, 34]. Thus, the objective of the current pilot study was to examine acute serum hormone levels for young women (ages 18-35) presenting at a hospital emergency department (ED) with symptoms of TBI and to compare these levels with those of healthy female controls matched for age while controlling for OC status. We did not include a trauma control group as we wanted to obtain true baseline comparisons that would not be obtainable in a polytrauma-only control group and to allow us to better delineate effects of oral contraceptives on the hormone levels. As noted by Wagner et al. [16], the literature [35–37] documents that peripheral aromatization has been implicated in the stress-induced increases in estradiol levels for individuals with polytrauma. Addition of a polytrauma control group would have made the current study objectives difficult to attain.

Based on existing TBI research findings, we hypothesized that

1. Hormone trajectories would be significantly different between women with TBI (cases) and controls
2. Fluctuations in hormone levels over 72 hours (i.e., slopes of the trajectories) would differ for cases and controls and with key covariates such as OC use and injury severity

## 2. Methods

### 2.1. Participants and Procedure

The Carolinas HealthCare System Institutional Review Board approved the study protocol, and the consent allowed for proxy consent if the potential participant was unconscious or sedated. Study personnel screened ED admissions for women meeting the study criteria. Women were approached for study enrollment in the ED using not only the documented diagnosis of a mild to severe TBI in the medical record (MR) by the ED admitting physician but also by accompanying MR terminology reflecting the clinical signs of mild to severe TBI presented in the Department of Veterans Affairs (VA) and Department of Defense (DoD) Clinical Practice Guidelines [38]. Not all women presenting with symptoms were given a CT scan. The following VA/DoD definition of TBI and symptom list were thus also used to guide study staff for screening and enrollment in the ED should potential participants have mild symptoms of TBI [45]: “Traumatic brain injury is a traumatically induced structural injury and/or physiological disruption of brain function as a result of an external force … indicated by new onset or worsening of at least one of the following clinical signs immediately following the event: any period of loss of or a decreased level of consciousness, any loss of memory for events immediately before or after the injury (posttraumatic amnesia), any alteration in mental state at the time of the injury (e.g., confusion, disorientation, slowed thinking, and alteration of consciousness/mental state), neurological deficits (e.g., weakness, loss of balance, change in vision, praxis, paresis/plegia, sensory loss, and aphasia) that may or may not be transient, and intracranial lesion.”

Participants were 21 women presenting with TBI symptoms at a Level 1 Trauma Center ED and 21 healthy controls who were nurses on the inpatient obstetrical unit. Both groups ranged in age from 18 to 35. Enrollment occurred from March of 2013 to June of 2015. Inclusion criteria for both women with TBI and controls were (a) age between 18-35 and (b) for the women with TBI, presentation at the ED with TBI, as diagnosed by the admitting physician from CT scan, symptoms reported, or both as well as the study definition adopted from the VA/DoD Guidelines [38] for diagnosis of mild TBI. Exclusion criteria were (a) being pregnant, which was determined for all women of childbearing age at the time of ED admission through a pregnancy test, and (b) prior TBI, for both women with TBI and controls. Healthy controls had 20 mL of blood taken at time of consent and 72 hours later. 80% of the total enrolled cases had mild TBI, which is consistent with the general epidemiological breakdown of TBI severity [39]. We did use proxies for consent and information such as menstrual history, prior TBI, and reproductive information with our severely injured participants. While this was a small portion of our total sample, proxies may have occasionally reported inaccurate information. For this reason, an OB/GYN fertility physician study coinvestigator determined menstrual phase of each participant from the hormone levels in our serum blood samples. While pregnancy tests are routinely completed for women with TBI in the ED, our controls also self-reported whether or not they were pregnant and the physician also determined menstrual phase for our controls. Therefore, while an actual pregnancy test would have been important for the controls, the physician was able to determine whether women were pregnant by hormone levels in the blood samples.

### 2.2. Measures

#### 2.2.1. Demographic and Reproductive Information

Information was collected from participants, or by proxy, as well as the medical record regarding age, marital status, level of education, and medical and mental health history. A menstrual history questionnaire was also completed regarding premorbid use and type of OCs. TBI history and current injury information were collected on participants with TBI via interview with the patient or family member and a review of the history and physical in the patient electronic medical record. Time of admission and initial blood samples were recorded in the ED and in our study log. Most samples were drawn within a few minutes or up to 3 hours after admission both in the ED and trauma surgery.

#### 2.2.2. Glasgow Coma Scale (GCS)

The GCS [40] is a standard method for determining injury severity that evaluates eye opening, verbal response, and motor response. Admission GCS scores ranged from 3 to 15; those with GCS 13-15 were categorized as having mild TBI, those with scores of 9-12 were categorized as having moderate injury, and those with GCS scores of 3-8 were categorized as having severe injury. In addition to recording of the ED use of GCS scores for all of our participants with TBI, the DoD case definition for mild TBI was used for enrollment purposes [38]. While the GCS remains the gold standard measure of injury severity [40], the DoD case definition is useful, particularly when describing mild TBI characteristics.

#### 2.2.3. Hormone Levels

For individuals with TBI, reproductive hormone levels of estradiol, progesterone, LH, and FSH as well as cortisol levels were obtained and processed from blood samples at time of ED admission and within 72 hours of admission. Control hormone levels were collected through blood samples taken following consent and at 72 hours after enrollment. All sample analyses were done by enzyme-linked immunosorbent assay (ELISA).

Blood samples for women with TBI and controls were collected at time of admission to the ED for treatment of injury and 72 hours later in the acute postinjury phase in BD Vacutainer® serum separator tubes (BD Biosciences, San Jose, CA). Upon collection, each sample was centrifuged at 1000xg, aliquoted, and stored at -80°C until the time of assay.

#### 2.2.4. Cortisol

Serum cortisol concentration measurements were done using an ELISA, per manufacturer's instruction (Enzo Life Sciences LTD, UK). The interassay and intra-assay coefficients of variance (CVs) were < 10%, and samples that fell below the detection limit were assigned the value of the detection limit of the assay.

#### 2.2.5. Sex Hormones

Estradiol, progesterone, LH, and FSH were all analyzed on Beckman Coulter DXI using competitive binding ELISA assays (Siemens Healthcare Diagnostics, 511 Benedict Avenue, Tarrytown, NY) following the manufacturer's instructions. The amount of analyte in each sample was determined from a stored, 6-point linear calibration curve. Interassay and intra-assay coefficients of variance (CVs) were < 10%, and any samples that fell below the detection limit were assigned the value of the detection limit of the assay.

#### 2.2.6. Menstrual Cycle Phase

For women with TBI and controls, the obstetrician/gynecologist fertility physician investigator (BSH) determined menstrual cycle phase from serum sample levels as well as self- or proxy-reported date of last menstrual period.

### 2.3. Data Analyses

Mean, medians, percents, and counts of hormone levels as well as demographic, medical, menstrual history and injury-related information for the women with TBI were calculated and graphed. Standard error of the mean was used. To determine whether women with TBI and controls differed in demographic characteristics, two analyses of variance (ANOVAs) were run to assess education level and age. Five hierarchical linear models (HLMs) were then performed to examine whether linear trajectories of the five hormones (estradiol, FSH, LH, progesterone, and cortisol) at baseline and the time 1 (72-hour follow-up) differed between controls and women with TBI. Counts and percentages were also calculated related to the time of injury, severity of injury, and OC use. A power analysis was performed using G^∗^Power 3 in order to determine the effect sizes that a comparison between two groups over two time points could detect with the current sample size. With 80% power (1-*β*) and assuming a correlation between repeated measures at *r* = 0.60, a sample size of 42 participants is enough to detect all large-sized effects (>*f* = 0.40) but no medium- or small-sized effects. As a result, null comparisons over time between women with and without TBI should be interpreted with an appropriate degree of caution given the pilot nature of this sample.

Injury group (control vs. TBI), time, and the group^∗^time interaction terms were all entered simultaneously as fixed effects into the HLMs. Hormone levels at each of the two time points were entered into the five HLMs as the dependent variable. Statistically significant fixed effects of injury group on hormone trajectories were then graphed across each of the two time points. Main effects indicate hormone levels over 72 hours varied as a function of the predictor variable, and significant interaction effects were examined.

For the TBI group only, HLMs were performed to examine whether linear trajectories of the five hormones at baseline and the time 1 follow-up differed as a function of GCS score and (OC) use at baseline by women with TBI. Given the consistent finding with the HLMs showing no effect of time on hormone levels or interactions with severity, OC use, and hormone levels, hormone levels for time 1 and time 2 were averaged and linear regression models run to determine the independent effects of OC use and injury severity on hormone levels after TBI.

## 3. Results

### 3.1. Baseline Demographics and Reproductive History

Tables 1(a) and 1(b) present baseline demographics and menstrual/reproductive history of women with TBI and controls.

There were no significant differences between women with TBI and controls regarding the number of days between cycles. Of the entire sample (*N* = 42), most women reported averaging 24-35 days (66.7%) between periods, with 19% reporting 36-90 days between periods, and 14.3% reporting below or above the upper (90 days) and lower (24 days) limits. Thirty-eight percent of both study groups were taking oral contraceptives (OCs). Women with TBI and controls differed significantly with regard to educational level. At the time of injury (or enrollment for controls), only four women were in the luteal phase of their menstrual cycle. Also, eight women in each group were using OC at the time of injury or enrollment. Years of education ranged from 9 to 16 for women with TBI but from 12 to 16 years for controls. Mean age was also significantly different for the two groups (TBI 25.81, control = 29.52; *p* = 0.010). As a result, all HLMs comparing the two groups' hormone levels over 72 hours were controlled for education and age.

### 3.2. Effects of Injury Status on Sex Hormone and Cortisol Levels

Statistically significant and nonsignificant fixed effects from the first set of HLMs, as well as their b-weights and *p* values, appear in Table 2.

**Figure 1:**
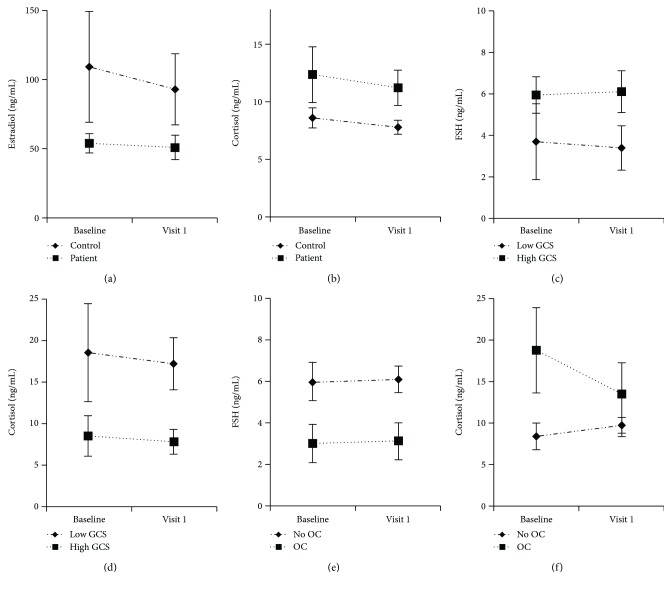
(a) Effect of group on estradiol trajectories with standard error bars, *N* = 42. (b) Effect of group on cortisol trajectories with standard error bars, *N* = 42. (c) Effect of GCS on FSH trajectories with standard error bars, *N* = 21. (d) Effect of GCS on cortisol trajectories with standard error bars, *N* = 21. (e) Effect of OC on FSH trajectories with standard error bars, *N* = 21. (f) Effect of OC on cortisol trajectories with standard error bars, *N* = 21.

In the HLM with estradiol trajectories, after controlling for age and education, there was a significant main effect for injury status (*p* = 0.042), but no time^∗^group interaction (*p* = .639), suggesting higher estradiol levels among controls compared to cases across the two time points (Figure 1(a)) and estradiol suppression due to TBI. In the HLM with cortisol trajectories, after controlling for age and education, there was a significant main effect for group (*p* = 0.028), but no time^∗^group interaction (*p* = .863), showing that individuals with TBI had higher cortisol levels than controls from time one to time 2 (see Figure 1(b)).

In the HLMs with remaining hormone trajectories, after controlling for covariates, there were no main effects for group (all *p*s ≥ 0.282) and no time^∗^group interactions (all *p*s ≥ 0.511).

### 3.3. Effects of Injury Severity on Reproductive Hormones and Cortisol

We then modeled the effects of injury severity (GCS) on hormones for individuals with TBI only with GCS, time, and the GCS^∗^time interaction term as the independent variables (Table 3).

The HLM assessing GCS and FSH trajectories showed a significant effect for GCS (*p* = 0.021), but no significant time^∗^GCS interaction (*p* = .452), suggesting compared to controls, women with TBI with higher GCS scores had higher FSH levels over 72 hours (see Figure 1(c)). Also, there was a marginally significant effect for GCS on LH (*p* = 0.054), showing that women with TBI with more mild injury had marginally higher LH levels over 72 hours compared to women with lower GCS. The final significant effect for the GCS HLMs was on cortisol trajectories (*p* = 0.001), but there was no time^∗^GCS interaction (*p* = .391), indicating that cases with less severe TBI had lower cortisol levels from time 1 to time 2 blood draw when compared to women with more severe TBI (Figure 1(d)). There were no significant GCS effects or time^∗^GCS interaction effects with estradiol or progesterone trajectories.

### 3.4. Effects of Oral Contraceptives (OCs) on Sex Hormones and Cortisol

Additional regression models evaluated temporal and OC effects on hormone trajectories among individuals with TBI (see Table 4).

The HLM assessing OC status and FSH trajectories showed a significant effect for OC (*p* = 0.016), suggesting that women with TBI using OC at baseline had lower FSH levels from time 1 to time 2 than women who were not taking OCs at baseline Figure 1(e). In the HLM with cortisol trajectories, there was a significant main effect for OC (*p* = 0.008), showing these same women on OC at baseline also had higher cortisol levels from time 1 to time 2 blood draw than women with TBI who were not using OCs at baseline Figure 1(f). The HLMs with estradiol, LH, and progesterone trajectories showed no significant OC effects from time 1 to time 2 blood draw. Figures 1(a)–1(f) provides the graphic presentation of hormone findings obtained by the modeling group, hormones and GCS, and hormones and OCs.

After averaging time 1 and time 2 values for hormone levels, linear regression analyses showed significant associations between OC status and the interaction between injury severity and OC status, on both FSH and cortisol levels among women with TBI (Table 5).

**Figure 2:**
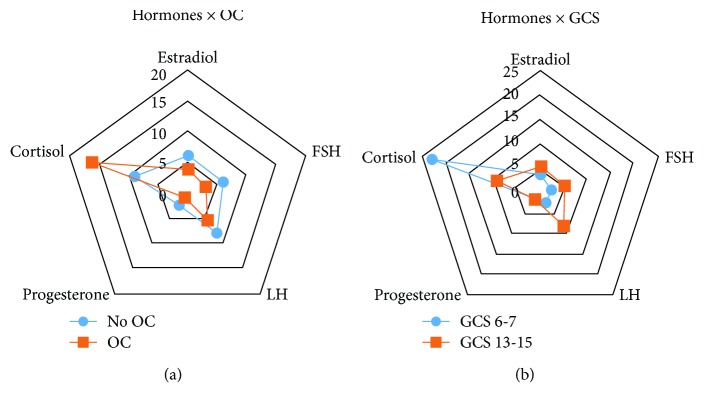
(a) Hormones by OC status, *N* = 21. Average baseline and follow-up hormone levels (ng/mL) in participants with TBI, categorized by OC usage. Estradiol levels were scaled by 0.1 to fit a 0-20 range. FSH levels differed significantly by OC status (*p* = 0.020). (b) Hormones by GCS, *N* = 21. Average baseline and follow-up hormone levels (ng/mL) in participants with severe and mild TBI. Estradiol levels were scaled by 0.1 to fit a 0-25 range. Cortisol levels were significantly different by GCS classification (*p* = 0.024).

These findings, also presented in Figures 2(a)–2(b), suggest that both injury severity and OC use at time of injury affect hormone levels.

## 4. Discussion

This pilot study compared acute sex hormone and cortisol trajectories in young women with TBI to healthy controls in an effort to illuminate complexities of endocrine responses for women after TBI. The specific investigation of young, premenopausal women, and the physiological effects of their OC use on endogenous hormone physiology, is a novel contribution to the literature. Though our study was small, our exclusions, such as limiting the age range of participants, uniquely controlled for variability in reproductive life stage and potentially wide-ranging effects on hormone levels at each of these stages. The findings of the study increase evidence of the complexities involved in adequately assessing the impact and association of sex and hormone physiology on the already heterogeneous acute response to TBI. Women sustain TBI at significantly lower rates than men [39], rendering this cohort a relatively large sample size from which to evaluate response to injury among women. However, the small sample size affected power for statistical modeling and limited the number of covariates explored to OC use and GCS. Given that both TBI and other trauma may contribute to similar hormonal derangements after injury, we used a healthy control group in this pilot study to characterize these derangements in a TBI population, regardless of additional injury type or TBI severity. Doing so now lays the groundwork to address questions like how much of the hormone changes observed are attributable to the systemic response to TBI vs. a systemic response to extracerebral trauma.

Most of the women with TBI had mild injuries (80%), and the findings add to the literature on TBI effects on acute hormone profiles published in a population with severe TBI [16, 41]. In the current study, greater injury severity was associated with reduced HPG function (i.e., lower FSH trajectories and marginally significant lower LH trajectories) and amplified HPA function (i.e. higher cortisol trajectories). These findings are similar to a study showing FSH/LH or gonadotrophic effects in a group of women patients with acute, primarily moderate to severe, TBI [41]. However, in the current study, compared to women with mild TBI not on OCs at baseline, women with mild TBI on OCs at baseline had lower FSH trajectories and higher cortisol trajectories from time 1 to time 2 blood draw. Our multivariate findings (Table 5) adjusting for OC status indicate that the stress of TBI independently impacts levels of circulating hormones and that impact increases as injury severity increases. These hormone profiles are similar to previous findings in severe TBI [16], which showed dramatic HPG axis shut down within the first 3-4 days after severe TBI as well as elevated cortisol levels for most study participants over the first week postinjury. This current study provides initial evidence that women with predominantly mild TBI, vs. with moderate to severe TBI, may also have significant hormonal changes in response to the injury, and these TBI effects increase as severity of injury increases. However, our investigations suggest that TBI effects on reproductive and stress hormone production could be compounded for women of childbearing age who are on synthetic hormones (OCs) at time of injury as both injury severity and OC status were independent predictors of cortisol and FSH.

TBI results in several acute secondary injury cascades documented in clinical populations with severe TBI, including aseptic inflammation [42, 43], excitotoxicity [44], monoaminergic dysfunction [45], neurotrophin abnormalities [46, 47], and ultimately CNS damage [48, 49]. There is a substantial body of experimental literature supporting the neuroprotective effects of progesterone and estradiol after TBI in mitigating damage due to secondary injury [11, 13, 15]. However, establishing if/how these relationships occur clinically has been a challenge. Central to this issue is that in addition to potential neuroprotective effects, clinical reports demonstrate progesterone has a major role in peripheral synthesis of sex (estradiol and testosterone) and stress (cortisol) hormones that are associated with poor outcome, particularly for older adults with TBI [16, 31].

In addition to being reflected in serum cortisol levels, high CNS levels are associated with poor long-term outcomes [31], and CNS cortisol has a complex regulatory influence on both inflammation and neurotrophin relationships to TBI outcome [49, 50]. Sex hormones like testosterone and estradiol are largely produced via extragonadal sources in the setting of severe TBI [16]. Individuals sustaining major polytrauma, even among those with relative mild TBI, experience significant peripheral steroidogenesis which may impact survival and outcome [51]. Further, sex hormones like estradiol have complex relationships with clinical TBI outcome, particularly mortality status, depending on their presence and concentration in the CNS [52] versus periphery [16]. Thus, future work should enroll clinical populations to begin to tease out endogenous hormone effects on secondary injury cascades, specifically for women after TBI given that experimental TBI models do not fully replicate the dual impact of critical illness and concurrent trauma on secondary injury cascades.

Previous work by Wunderle et al. [18] showed women with mild TBI and injured during the luteal phase of their menstrual cycle had significantly worse outcomes than women injured during the follicular phase of their cycle or women taking OCs. Gallagher et al. [33] also found that OC use in female college athletes with concussion resulted in less severe symptoms than women non-OC users. However, others have found no change in cognition or balance related to menstrual phase for young women athletes with concussion [32]. Yet, other work suggests menstrual cycle phase may not significantly alter HPA reactivity among women in the general population [53]. Based on work in severe TBI [16, 31], it is possible that elevated progesterone levels during the luteal phase of the menstrual cycle contribute to systemic increases in serum estradiol, testosterone, or cortisol levels that may negatively impact outcome. Future work with larger sample sizes should be pursued to examine (1) how menstrual cycle phase affects peripheral steroidogenesis and outcome for women over a range of TBI severity and (2) how progesterone supplementation/therapy specifically influences peripheral steroidogenesis given the null results from the two recent phase III multisite clinical trials [14, 30].

Studies demonstrate OC use has significant effects on baseline adrenal function as well as adrenal activity with adrenocorticotropic hormone (ACTH) stimulation [54]. This work has implications for TBI, wherein women on OCs may have an amplified cortisol response after TBI. While the current study is underpowered to conduct this analysis, the fact that injury severity and OC use each carried independent effects on acute cortisol levels supports the need for future research to assess this possibility. Despite this possibility of amplified HPA reactivity among OC users, other studies involving persons with mild TBI do support the idea that OC use may have beneficial effects on outcome [55] and suggest larger studies are needed to delineate OC effects on TBI outcomes across the injury severity spectrum.

When considering the potential impact that OC use can have on outcome, it is notable that OC use can reportedly increase chronic inflammation [56] as well as affect monocyte-derived macrophage function and DNA methylation [57]. Given recent evidence from our group and others [58–60] that chronic inflammation occurs after TBI, future work should focus on the moderating effects of OC status (at the time of injury and postinjury) on posttraumatic inflammation and its downstream effects on CNS damage, neurodegeneration [59, 61], and TBI related complications [62–65]. DNA methylation in brain tissue has been observed in animal models of TBI [66], and many genes involved in secondary injury cascades after TBI are regulated through epigenetic mechanisms [67]. Thus, future work might also explore how OC use may moderate epigenetic effects on TBI pathophysiology and outcomes.

Our work in this report focused on younger premenopausal women. However, previous work suggests HPG axis shut down for menopausal as well as premenopausal women that is affected by systemic cortisol levels after TBI [12]. Future work might consider how menopause, as well as various synthetic and natural hormone replacement therapies, influences sex hormone physiology in both the acute and chronic phases of TBI recovery as well as TBI outcomes.

### 4.1. Study Limitations

While it is a pilot and exploratory, the small N leads us to adopt a tentative and cautious tone for our report of findings, discussion, and conclusions. Second, our control group was not specifically matched for age and education. However, we uniquely matched our study groups in terms of the age range of persons enrolled to control for variability in sex hormone levels due to wide ranges in reproductive life phases in our sample. Third, not all of our participants with mild TBI diagnoses were given CT scans on admission. While all were diagnosed with concussion by the ED physician based on symptoms at admission and had a GCS of 13-15, there is a possibility that some may have not sustained a TBI. Fourth, the significantly higher level of education for the controls vs. women with TBI may have affected the results in terms of potentially poorer prestudy and preinjury overall, nutrition, health, and cognition. Fifth, we did not have a trauma or critical illness control group; we uniquely chose healthy women 18-35. While we do not conclude that hormone changes are specifically/only due to the TBI, these hormonal derangements observed (regardless of etiology) may be relevant to recovery among women with TBI.

It is also important to note that the TBI itself may be an important contributor to this response. The physiological response to TBI (including mild TBI) is leveraged in part via the autonomic nervous system which can impact HPA function [68–73]. Also, gonadotropin dysfunction has been documented with TBI across the range of injury severity [16, 74, 75]. Our results show that both TBI diagnosis and injury severity (based on GCS) influence cortisol and FSH levels; thus, there could be contributions that are TBI specific in addition to trauma in general. Avoidance of a potential confound through use of a healthy group of controls vs. a trauma control group allowed us to determine clearer baseline levels and effects of OCs. Sixth, we enrolled very few severely injured women. Power was thus insufficient to analyze the very few who may have differed from most in time to first blood draw, which, based on the ED log ranged from minutes to 3 hours. In future work, we acknowledge that we should develop a plan to track time to blood draw following admission to the ED as well as from time of injury and use these as covariates.

We also specifically chose to focus on sex and stress hormones in this pilot, though we are aware that there are other preliminary processes, as in the action of prolactin on gonadotropin-releasing hormone, that contribute to hormone secretion. Future research with a larger sample would allow sufficient power for examining how TBI's effects on sex hormones may be moderated by earlier and complex processes involved in secretion and for the addition of two other comparison groups of critical illness or extracerebral traumatic injury-only participants. Additionally, follow-up or multioccasion measurement of hormones will be important in future research. Finally, we did rely on family and significant others as proxies for the patient when unconscious or cognitively incapable to report accurate information. Proxy consent did not delay time to enrollment for this study; however, the possibility of delay will be a factor to consider when designing larger studies that include participants who need proxy consent. It is possible that these proxies could have provided inaccurate information.

## 5. Conclusions

The biological impact of sex and hormone physiology, regardless if from TBI and/or related extracerebral trauma, may influence psychosocial end-points to contribute to the complex biopsychosocial relationships associated with reported sex differences in TBI recovery. While we do not know if these changes are specifically or solely a result of the TBI, we believe that our findings provide a step toward further research and discovery that can inform this question. The current report also does not evaluate if/how acute hormones or OC status influence multidimensional outcomes. However, future work should address this point, such as through exploring OC-associated effects due to cortisol-binding globulin (CBG). Also, future research should examine possible mediation by precursors, like prolactin and gonadotropin-releasing hormone, to hormone secretion. Finally, future research should compare outcomes of critical illness, extracerebral trauma, and healthy control vs. trauma TBI participants, as well as conduct follow-up measurement of sex and stress hormones. Particular consideration should also be given to influences of innate sex differences in cognitive performance, as well as potential gender bias with functional and self/caregiver-reported outcome metrics. Our findings provide a step toward understanding responses and effects of TBI and potentially toward targeted treatments for this devastating injury.

## Figures and Tables

**Table:** (a) Baseline participant with TBI demographics, menstrual cycle phase, and contraceptive use

Subject	Age	Education	Injury cause	GCS	ISS	Baseline menstrual phase	Contraceptive
1	18	12	Vehicular crash	15	0	Follicular	NA
2	27	14	Assault	15	0	Follicular	Lutera
3	23	15	Fall	13	12	Follicular	NA
4	31	12	Vehicular crash	7	30	Follicular	NA
5	30	9	Fall	15	0	Follicular	Depo
6	28	12	Vehicular crash	15	14	Follicular	NA
7	20	13	Vehicular crash	7	24	Follicular	^∗^BCP
8	31	13	Vehicular crash	15	0	Luteal	NA
9	19	10	Vehicular crash	14	12	Follicular	Depo
10	33	14	Vehicular crash	15	0	Follicular	NA
11	25	16	Vehicular crash	13	4	Follicular	Yasmin
12	31	13	Vehicular crash	15	5	Follicular	NA
13	29	15	Hit as pedestrian	15	1	Luteal	NA
14	33	12	Vehicular crash	15	5	Follicular	NA
15	20	13	Vehicular crash	13	9	Luteal	NA
16	21	13	Hit as pedestrian	15	6	Follicular	NA
17	24	16	Hit as pedestrian	6	14	Follicular	Cryselle 28
18	33	12	Unknown	7	20	Luteal	NA
19	21	14	Fall	14	0	Follicular	NA
20	18	11	Unknown	15	14	Follicular	Depo
21	27	12	Vehicular crash	15	1	Follicular	Estradiol

^∗^Birth control pill: response of the family member of the unconscious participant. The family member did not know the name of the pill.

**Table:** (b) Baseline control demographics, menstrual cycle phase, and contraceptive use

Control	Age	Education	Injury cause	GCS	Baseline menstrual phase	Contraceptive
1	31	16	NA	NA	Follicular	Loestrin
2	30	16	NA	NA	Follicular	Loestrin
3	33	16	NA	NA	Follicular	NuvaRing
4	32	16	NA	NA	Luteal	NA
5	35	16	NA	NA	Follicular	NA
6	26	16	NA	NA	Follicular	NuvaRing
7	31	16	NA	NA	Follicular	NA
8	34	16	NA	NA	Follicular	NA
9	28	16	NA	NA	Luteal	NA
10	25	13	NA	NA	Follicular	NA
11	32	13	NA	NA	Follicular	Microgestin
12	32	16	NA	NA	Follicular	NA
13	28	16	NA	NA	Follicular	NA
14	32	13	NA	NA	Luteal	NA
15	24	13	NA	NA	Follicular	NA
16	27	13	NA	NA	Follicular	NA
17	27	16	NA	NA	Luteal	NA
18	24	16	NA	NA	Follicular	Orsythia
19	30	16	NA	NA	Follicular	NA
20	26	14	NA	NA	Follicular	Junel Fe
21	33	13	NA	NA	Follicular	Previfim

**Table 2:** Patient vs. control differences in hormone trajectories across baseline and time 1. *N* = 42.

Predictor variable	b weight	Std. error	df	*t*	*p* value	95% conf. interval	
						Upper	Lower
Estradiol							
Group		40.51	53.58	-2.08	**0.042** ^∗^	-165.51	-3.03
Follicle-stimulating hormone							
Group	-0.09	1.06	50.73	-0.08	0.935	-2.21	2.04
Luteinizing hormone							
Group	-2.35	2.16	63.30	-1.09	0.282	-6.67	1.97
Progesterone							
Group	-1.52	1.76	43.26	-0.86	0.394	-5.07	2.04
Cortisol							
Group	5.46	2.43	57.04	2.25	**0.028** ^∗^	0.60	10.32

Note. ^∗^*p* < 0.05; ^∗∗^*p* < 0.01. Models adjusted for time, group^∗^time interaction, education, and age.

**Table 3:** GCS Prediction of Hormone Trajectories across Baseline and Time 1 among individuals with TBI. *N* = 21.

Predictor variable	b weight	Std. error	df	*t*	*p* value	95% conf. interval	
						Upper	Lower
Estradiol							
GCS	0.46	2.19	24.38	0.21	0.834	-4.05	4.98
Follicle-stimulating hormone							
GCS	0.54	0.22	22.88	2.47	0.021^∗^	0.09	1.00
Luteinizing hormone							
GCS	0.75	0.37	22.15	2.03	0.054	-0.01	1.51
Progesterone							
GCS	-0.03	0.12	17.23	-0.25	0.809	-0.28	0.22
Cortisol							
GCS	-2.21	0.58	28.23	-3.83	0.001^∗∗^	-3.39	-1.03

Note. ^∗^*p* < 0.05; ^∗∗^*p* < 0.01; ^∗∗∗^*p* < 0.001. Models also adjusted for time and GCS^∗^time interaction.

**Table 4:** OC Prediction of hormone trajectories across baseline and time 1 among individuals with TBI. *N* = 21.

Predictor variable	b weight	Std. error	df	*t*	*p* value	95% confidence interval	
						Upper	Lower
Estradiol							
OC	-14.13	14.92	32.01	-0.95	0.35	-44.52	16.25
Follicle-stimulating hormone							
OC	-2.95	1.16	32.38	-2.54	0.016^∗^	-5.31	-0.59
Luteinizing hormone							
OC	-2.65	2.51	30.70	-1.06	0.299	-7.77	2.47
Progesterone							
OC	-0.99	1.26	23.08	-0.79	0.438	-3.59	1.61
Cortisol							
OC	10.34	3.64	32.55	2.84	0.008^∗∗^	2.93	17.75

Note. ^∗^*p* < 0.05; ^∗∗^*p* < 0.01; ^∗∗∗^*p* < 0.001. Models adjusted for time and time^∗^OC interaction.

**Table 5:** Linear regression models for hormones by OC status and GCS group after TBI averaging baseline and time 1. *N* = 21.

Model	*β*	Std. error	*t*	*p* value	95% CI	
					Lower	Upper
Estradiol						
Intercept	52.67	17.21	3.06	0.007^∗∗^	16.51	88.83
OC status	-21.59	14.19	-1.52	0.145	-51.42	8.22
GCS group	10.06	17.55	0.57	0.573	-26.81	46.94
FSH						
Intercept	3.86	1.20	3.20	0.005^∗∗^	1.33	6.39
OC status	-2.70	0.99	-2.72	0.014^∗^	-4.79	-0.61
GCS group	2.59	1.22	2.11	0.049^∗^	0.01	5.17
LH						
Intercept	3.16	2.68	1.17	0.254	-2.47	8.79
OC status	-1.80	2.21	-0.81	0.426	-6.45	2.84
GCS group	5.60	2.73	2.04	0.055	-0.14	11.34
Progesterone						
Intercept	2.07	1.61	1.28	0.216	-1.32	5.47
OC status	-1.34	1.33	-1.00	0.327	-4.15	1.45
GCS group	0.11	1.65	0.06	0.946	-3.35	3.58
Cortisol						
Intercept	19.90	3.00	6.62	<0.001^∗∗∗^	13.59	26.21
OC status	5.85	2.47	2.36	0.030^∗^	0.63	11.06
GCS group	-12.78	3.06	-4.17	0.001^∗∗^	-19.21	-6.34

Note. ^∗^*p* < 0.05; ^∗∗^*p* < 0.01; ^∗∗∗^*p* < 0.001. FSH: follicle-stimulating hormone; LH: luteinizing hormone.

## Data Availability

The data used to support the findings of this study are available from the corresponding author upon request.
